# Supplementary material for: Capillary constrictions prime cancer cell tumorigenicity through PIEZO1
Source: Nat Commun. 2025 Sep 1;16:8160. doi: 10.1038/s41467-025-63374-6 (PMC12402161; doi:10.1038/s41467-025-63374-6)
Supplement: Supplementary file 1 — Supplementary Information [file 41467_2025_63374_MOESM1_ESM.pdf]

rCapillary constrictions prime cancer cell tumorigenicity through PIEZO1

G. Silvani[1], C. Kopecky[2], S. Romanazzo[2], V. Rodriguez[3], A. Das[4], E. Pandzic[5], J. G. Lock[4], C Chaffer[3], K. Poole[6] and K. A. Kilian[1,2*]

1 School of Materials Science and Engineering, UNSW Sydney, Australia
2 School of Chemistry, Australian Centre for Nanomedicine (ACN), UNSW Sydney, Australia
3 Garvan Institute of Medical Research, Darlinghurst, NSW 2010, Australia
4 School of Biomedical Sciences, Faculty of Medicine and Health, UNSW Sydney, Australia
5 Katharina Gaus Light Microscopy Facility, Mark Wainwright Analytical Centre, UNSW, Sydney, Australia
6 School of Biomedical Sciences, Faculty of Medicine & Health, UNSW Sydney, Australia
Corresponding author: k.kilian@unsw.edu.au

Supplementary Material

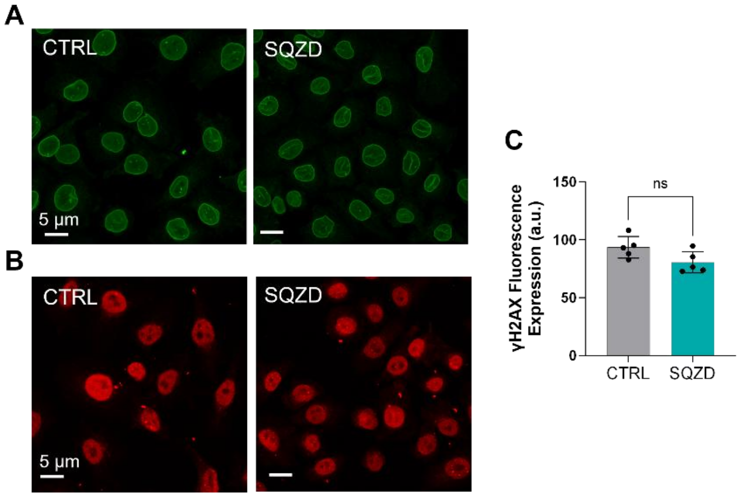

Supplementary Figure 1 No significant differences in nuclear structure and DNA damage were observed between control and squeezed conditions. (A) Representative staining of nuclear protein Lamin A in CTRL and SQZD melanoma cells. **(B)** and **(C)** Representative staining of γH2A.X in CTRL and SQZD melanoma cells. Bar graph displaying the mean fluorescence levels across 5 independent replicates per condition (≥20 cells per replicates). Bars represent mean ± SEM. Statistical significance was assessed using two-sided unpaired t-tests. p = 0.0606 (ns), 95% CI: [-26.57, 0.7287].

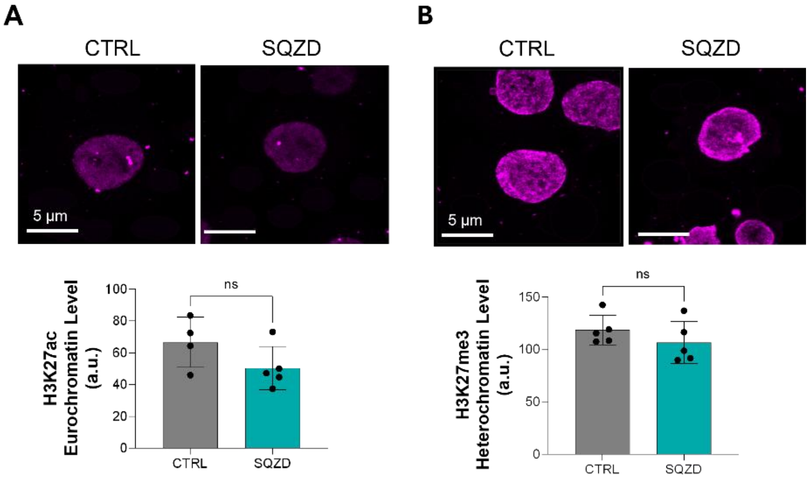

Supplementary Figure 2 No significant differences in H3K27 acetylation or trimethylation were observed between control and squeezed conditions. (A) Representative staining of H3K27ac in CTRL and SQZD melanoma cells. Bar graph displaying the H3K27ac levels in CTRL and SQZD cells. **(B)** Representative staining of H3K27m3 in CTRL and SQZD melanoma cells. Bar graph displaying the H3K9me3 levels in CTRL and SQZD cells. All bar graph displaying the mean fluorescence levels in CTRL and SQZD melanoma cells across 5 independent replicates per condition (≥20 cells per replicates). Bars represent mean ± SEM. Statistical significance was assessed using two-sided unpaired t-tests. H3K27ac p = 0.1425 (ns), 95% CI: [-39.17, 6.946]; H3K27me3 p = 0.3065 (ns), 95% CI: [-37.05, 13.23].

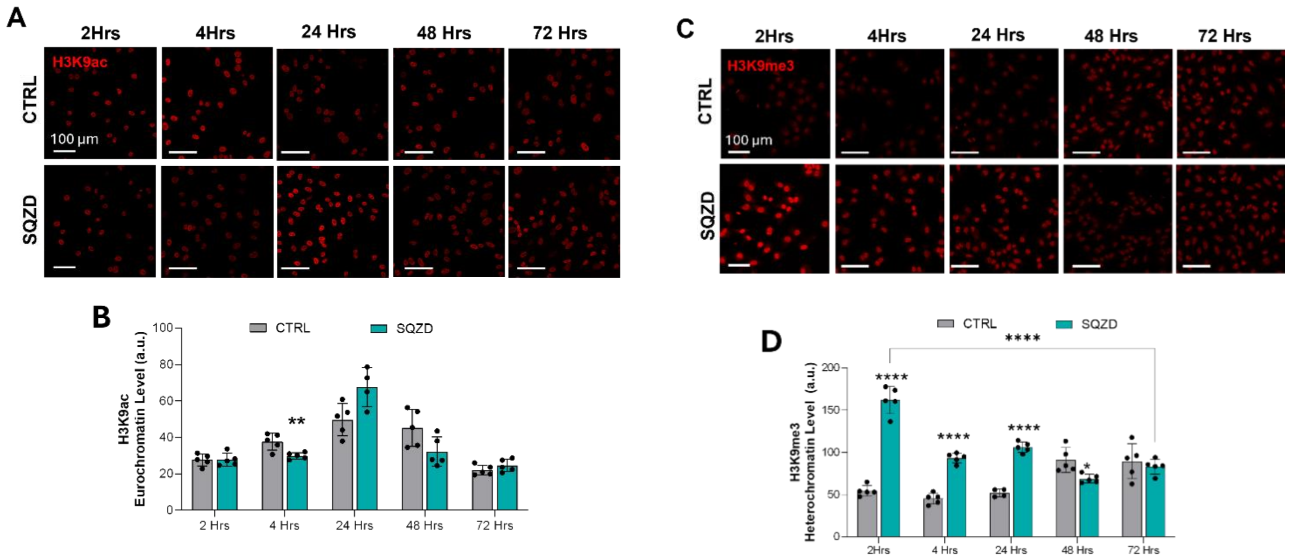

Supplementary Figure 3 Temporal dynamics of histone modifications following mechanical deformation. Representative immunofluorescence images of H3K9ac **(A)** and H3K9me3 **(C)** staining at different times post-constriction in control (CTRL) and squeezed (SQZD) A375-MA2 cells. Bar graphs displaying the quantification of ABCB5 H3K9ac **(B)** and H3K9me3 **(D)** mean fluorescence intensity levels at multiple time points (4 hrs to 72 hrs) and across 5 independent replicates per condition (≥20 cells per replicates). Bars represent mean ± SEM. Statistical significance was assessed using one-way ANOVA (two-sided). Asterisks indicate p-values, as follow: H3K27ac, 4Hrs $p = 0.0078$ (**), 95% CI: [-12.80, -2.667]; H3K27me3, 2Hrs $p < 0.0001$ (****), 95% CI: [89.50, 125.2]; H3K27me3, 4Hrs $p < 0.0001$ (****), 95% CI: [39.17, 57.20]; H3K27me3, 24 Hrs $p < 0.0001$ (****), 95% CI: [45.45, 62.93]; H3K27me3, 2Hrs Vs 72Hrs $p < 0.0001$ (****), 95% CI: [-97.62, -59.59].

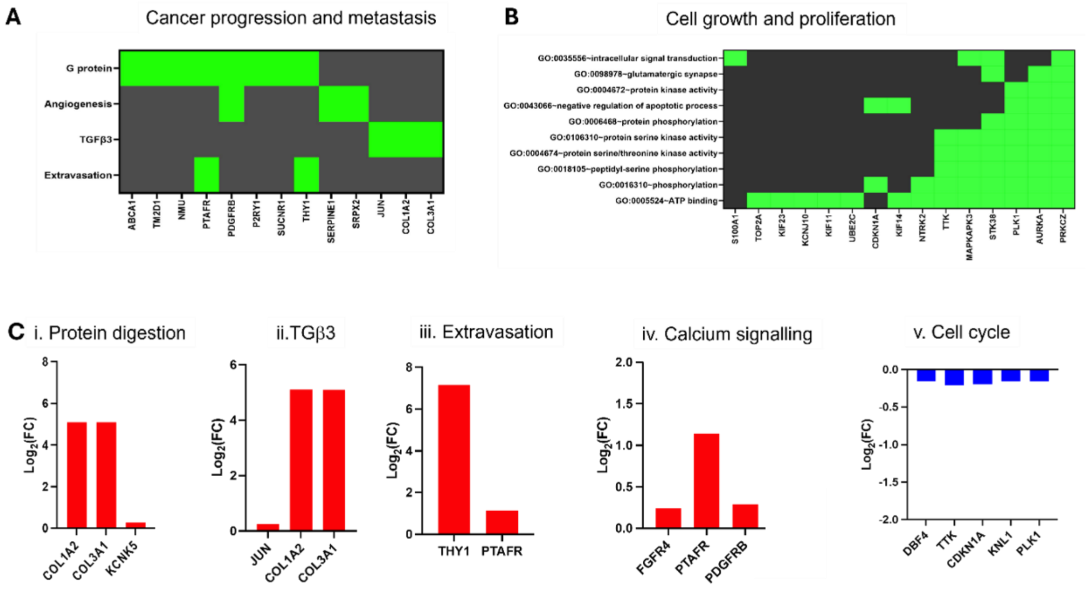

Supplementary Figure 4 Gene ontology analysis. (A, B) Enriched functional annotations for biological processes involved in cancer progression and metastasis and cell proliferation activity. **(C)** Bar graphs showing the average LogFoldChange (LogFC) between SQZD and CTRL conditions ($n = 3$ biological samples) for genes involved in: (i) protein digestion, (ii) TGF-β3 signalling, (iii) extravasation, (iv) calcium signalling, and (v) cell cycle pathways.

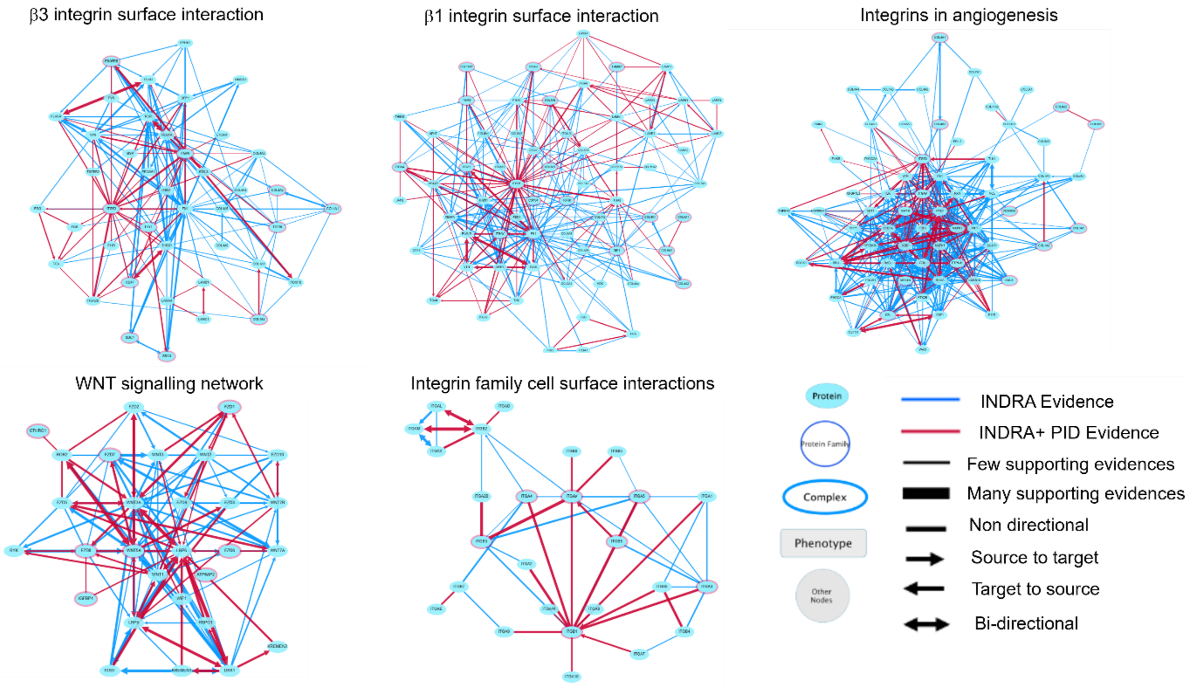

Supplementary Figure 5 Analysis of gene function and construction of functional networks. Gene networks representation derived from genes upregulated (p<0.025) (derived from RNAseq analysis of CTRL and SQZD (n=3)) , fed to Cytoscape and analysed by KEGG database. Upregulated genes recognised in the different networks, were marked in red. INDRA: Integrated Network and Dynamical Reasoning Assembler; PID: Pathway interaction database.

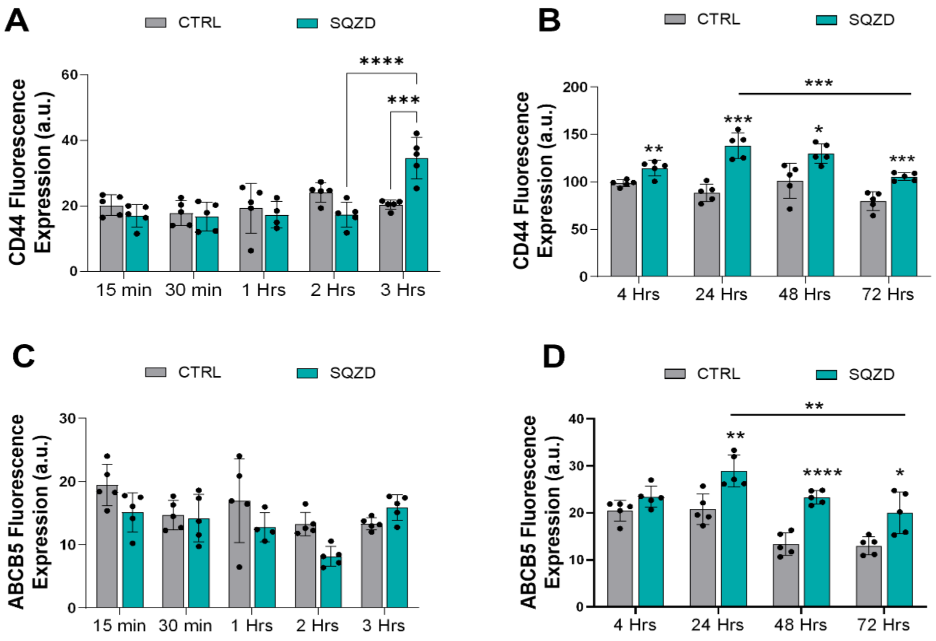

Supplementary Figure 6 Temporal dynamics of phenotypic marker expression following transient mechanical constriction. **(A)** and **(B)** Bar graph displaying the CD44 levels in CTRL and SQZD cells at early (15 min to 3 hrs) and late (4 hrs to 72 hrs) time points. **(C)** and **(D)** Bar graphs displaying the ABCB5 levels in CTRL and SQZD cells at early (15 min to 3 hrs) and late (4 hrs to 72 hrs) time points. All bar graph displaying the mean fluorescence levels in CTRL and SQZD melanoma cells across 5 independent replicates per condition (≥20 cells per replicates). Bars represent mean ± SEM. Statistical significance was assessed using one-way ANOVA (two-sided). Asterisks indicate p-values, as follow: CD44, 2HRS SQZD Vs 3HRS SQZD p <0.0001 (****), 95% CI: [-26.70, -7.914]; 3HRS CTRL Vs 3HRS SQZD p <0.0001 (****), 95% CI: [-23.72, -4.93126.70 to -7.914]. CD44 4HRS p = 0.0040 (**), 95% CI: [6.725, 25.14]; 24 HRS p = 0.0001 (***), 95% CI: [32.95, 67.01]; 48 HRS p = 0.0154 (*), 95% CI: [7.20, .68]; 72 HRS p = 0.0006 (***), 95% CI: [14.72, 36.65]. ABCB5 24HRS p = 0.0040 (**), 95% CI: [3.309, 13.04]; 48HRS p <0.0001 (****), 95% CI: [7.054, 12.90]; 72HRS p = 0.0115 (*), 95% CI: [2.058, 12.00].

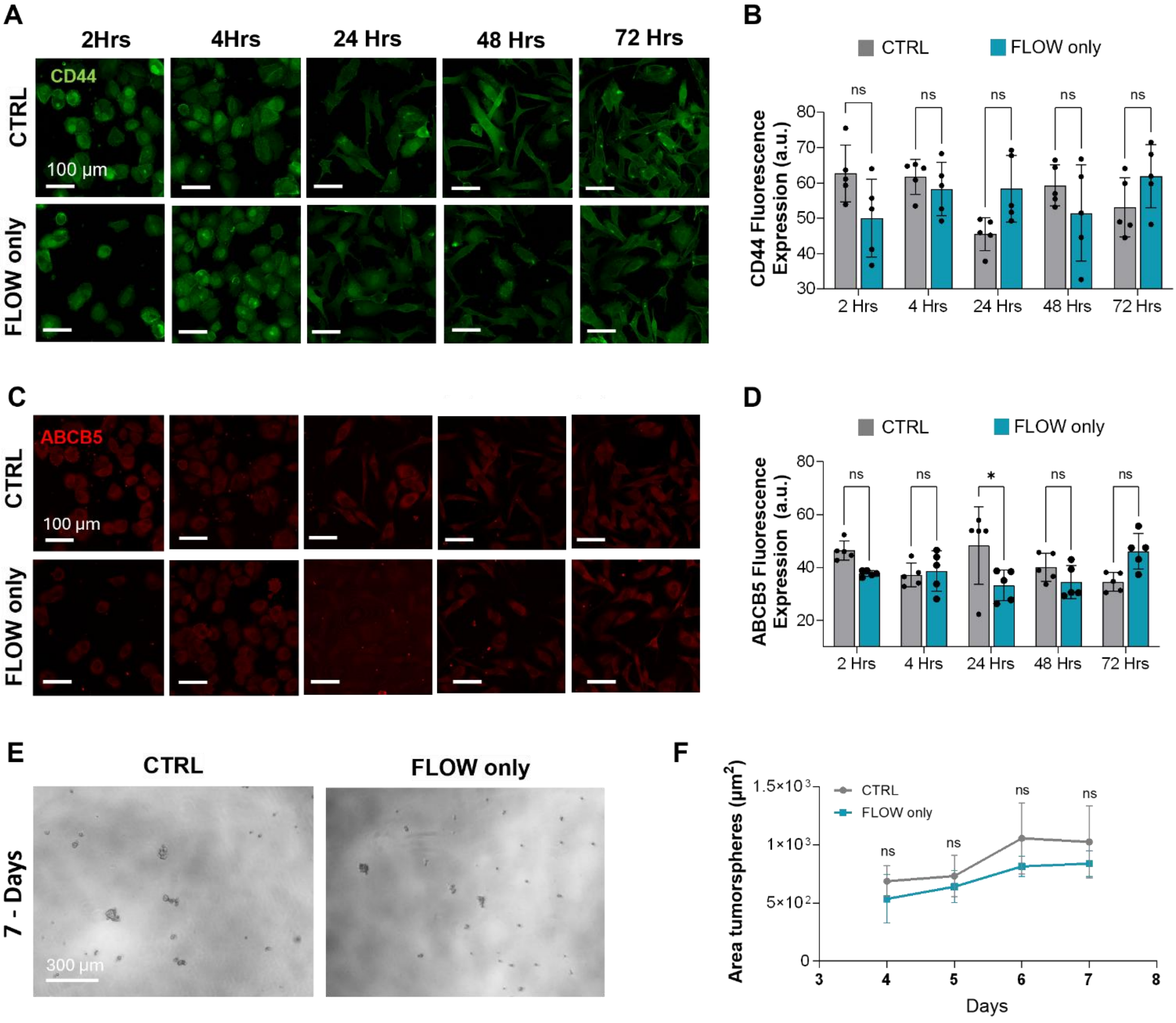

Supplementary Figure 7 Mechanical deformation, not flow-induced shear stress, drives acquisition of stem-like traits. Immunofluorescence analysis of CD44 (green, **A**) and ABCB5 (red, **C**) expression in control (static culture) and flow-only (no constriction) conditions. **(B, D)** Bar graphs displaying quantification of CD44 and ABCB5 fluorescence intensity across 5 independent replicates per condition (≥20 cells per replicates). Bars represent mean ± SEM. Statistical significance was assessed using one-way ANOVA (two-sided). Asterisks indicate p-values, as follow: CD44, 2HRS p = 0.4070 (ns), 95% CI: [-5.705 to 30.99]; 4HRS p = 0.9998 (ns), 95% CI: [-14.95, 21.74]; 24HRS p = 0.3893 (ns), 95% CI: [-31.15, 5.542]; 72HRS p = 0.8370 (ns), 95% CI: [-27.15, 9.540]. ABCB5, 2HRS p = 0.6041 (ns), 95% CI: [-5.829, 23.12]; 4HRS p >0.9999 (ns), 95% CI: [-15.95, 13.00]; 24HRS p = 0.0356 (*), 95% CI: [0.5859, 29.54]; 48 HRS p = 0.9538 (ns), 95% CI: [-8.967 to 19.98]; 72 HRS p = 0.2090 (ns), 95% CI: [-26.14 to 2.809]. **(E)** Representative brightfield images of tumorsphere formed from control and flow-only groups at day 7. **(F)** Tumorsphere growth curves over time, showing no significant differences between control and flow-only conditions, n = 3 independent replicates per condition. Bars represent mean ± SEM. Statistical significance was assessed using one-way ANOVA (two-sided). CTRL Vs FLOW only: 4-day, p = 0.3504 (ns) 95% CI [-549.1 to 246.4]; 5-day p = 0.5203 (ns) 95% CI -454.5 to 270.6]; 6-day, p = 0.2573 (ns) 95% CI [-749.7 to 266.6]; 7-day, p = 0.3814 (ns) 95% CI [-711.4 to 339.5].

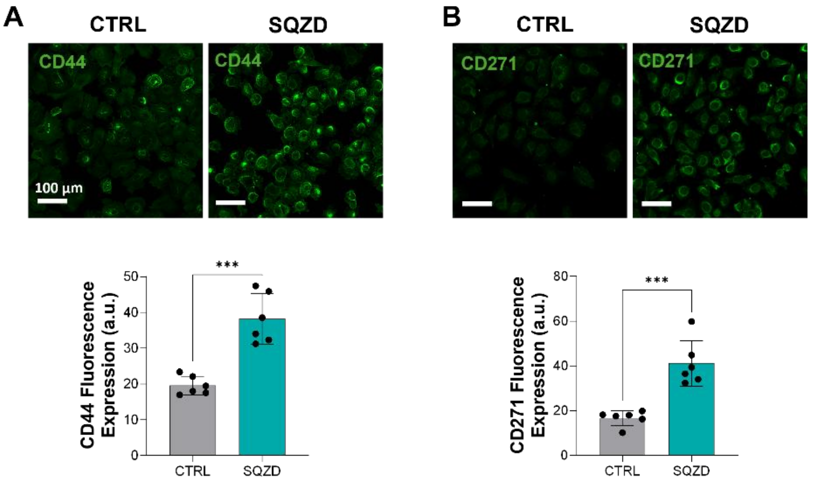

Supplementary Figure 8 Verification of elevated tumorigenicity and stemness marker expression in A375-luciferase melanoma cells prior to in vivo experiments. (A) Representative staining of CD44 in CTRL and SQZD melanoma cells. Bar graph displaying the mean fluorescence levels in CTRL and SQZD melanoma cells, across 6 independent replicates per condition (≥20 cells per replicates). Bars represent mean ± SEM. Statistical significance was assessed using two-sided unpaired t-tests. Statistical significance was assessed using two-sided unpaired t-tests. Asterisks indicate p = 0.0001 (***), 95% CI: [11.84, 25.44]. **(B)** Representative staining of CD271 in CTRL and SQZD melanoma cells. Bar graph displaying the mean fluorescence levels in CTRL and SQZD melanoma cells across 6 independent replicates per condition (≥20 cells per replicates). Bars represent mean ± SEM. Statistical significance was assessed using two-sided unpaired t-tests. Statistical significance was assessed using two-sided unpaired t-tests. Asterisks indicate p = 0.0002 (***), 95% CI: [14.72, .34.17].

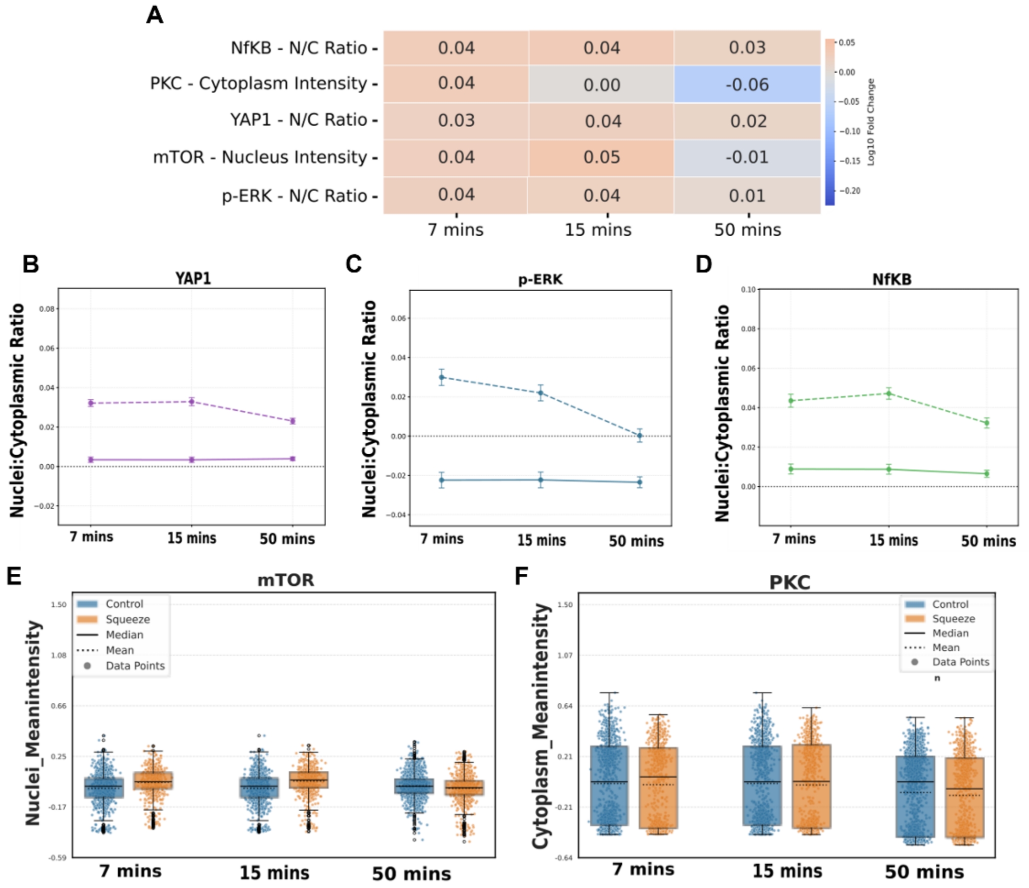

Supplementary Figure 9. Phosphoprotein imaging reveals time-resolved activation of mechanosensitive signalling pathways. (A) Heatmap representing $\log_{10}$ fold change in the activation of selected phospho-markers (NF-κB, PKC, YAP1, mTOR, and p-ERK) at 7-, 15-, and 50-minutes post-constriction. Line plot showing quantification of N/C ratios for YAP1 **(B)**, p-ERK **(C)**, and NF-κB **(D)** over time (average ± SEM represented for n ≥250 cells). Dash line represents SQZD sample while solid line represents CTRL group. The observed trend was confirmed with 2x repeat experiments. Box plot representing mTOR nuclear intensity **(E)** and PKC cytoplasmic intensity **(F)** over time (n ≥250 cells). The observed trend was confirmed with 2x repeat experiments.

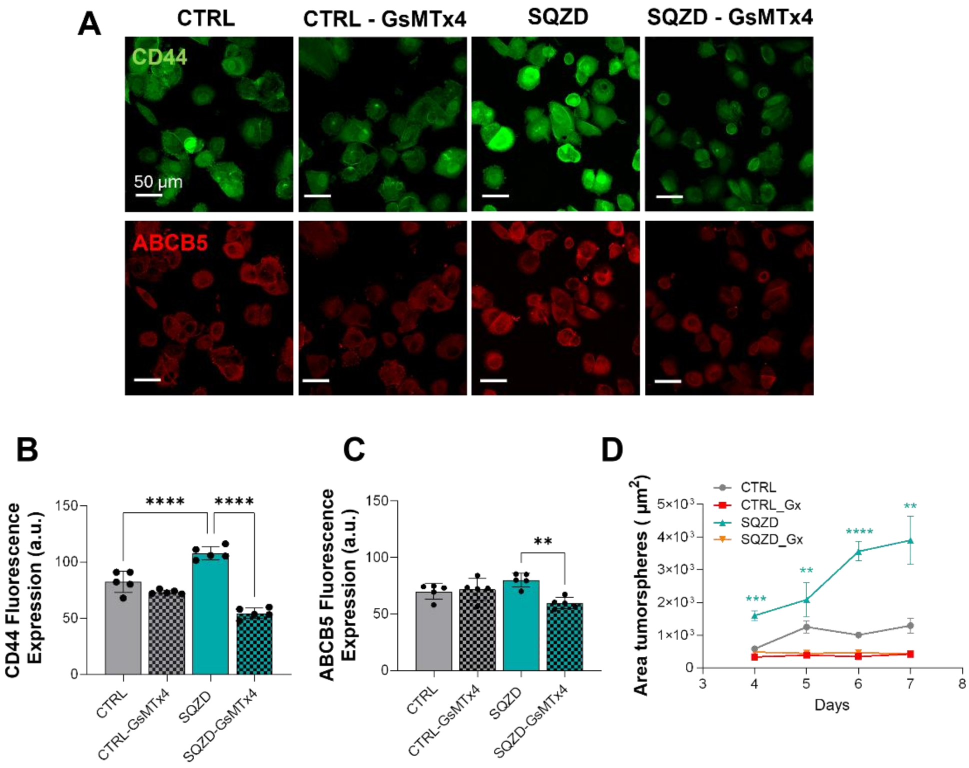

Supplementary Figure 10 Inhibition of mechanosensitive channels with GsMTx4 reduces stemness marker expression and tumorsphere formation following mechanical squeezing. (A) Representative immunofluorescence images of CD44 and ABCB5 expression in control (CTRL), squeezed (SQZD), and both cells group treated with GsMTx4. **(B, C)** Bar Graph displaying quantification of fluorescence intensity for CD44 and ABCB5 across experimental groups. Bar graph displaying the mean fluorescence levels across 5 independent replicates per condition (≥20 cells per replicates). Bars represent mean ± SEM. Statistical significance was assessed using one-way ANOVA (two-sided). Asterisks indicate p-values, as follow: CD44, CTRL Vs SQZD p <0.0001 (****) 95% CI: [-36.53, -14.43]; SQZD Vs SQZD-GsMTx4 p <0.0001 (****) 95% CI: [42.51, 64.61]. ABCB5, SQZD Vs SQZD-GsMTx4 p = 0.0018 (**) 95% CI: [7.466, 33.44]. **(D)** Tumorsphere growth curves over time showing no significant differences between control and flow-only conditions, n = 3 independent replicates per condition. Bars represent mean ± SEM. Statistical significance was assessed using a two-sided unpaired t-test. SQZD Vs SQZD_Gx, 4-day p=0.0003 (***) 95% CI [-1376 to -853.8]; 5-day p=0.0059 (**) 95% CI [-2487 to -789.2]; 6-day p<0.0001 (****) 95% CI [-3565 to -2625]; 7-day p=0.0013 (**) 95% CI [-4649 to -2279].

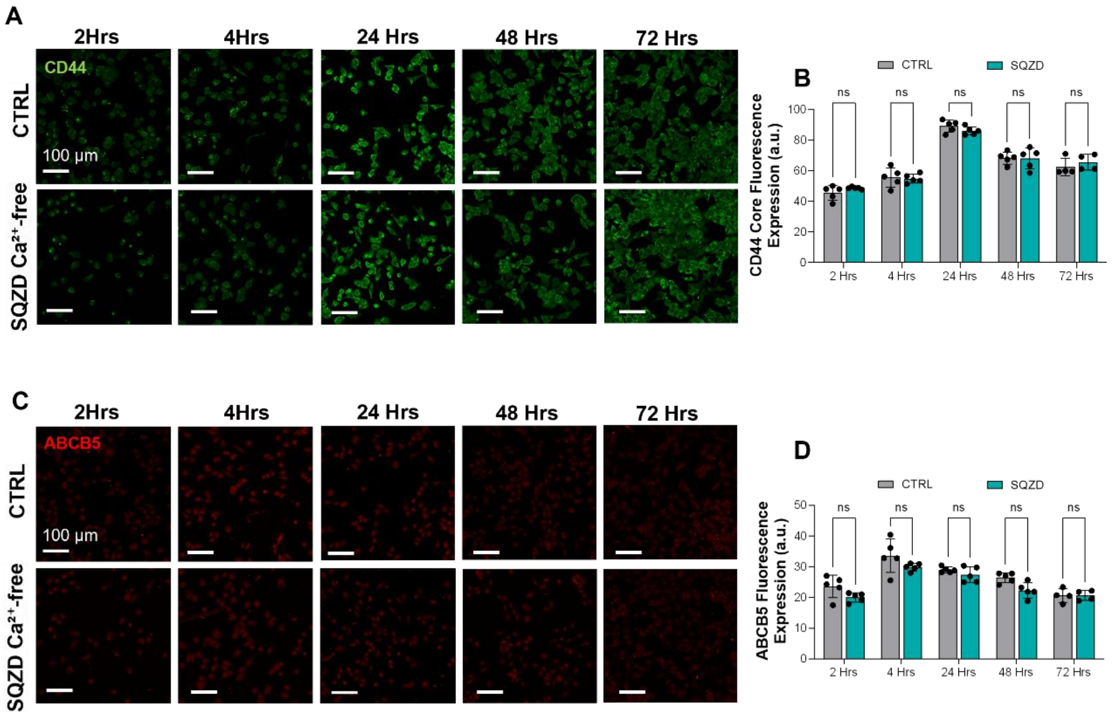

Supplementary Figure 11 PIEZO1-induced stemness marker expression is dependent on extracellular calcium influx. Representative immunofluorescence images showing CD44 **(A)** and ABCB5 **(C)** expression in melanoma cells under different conditions: control (CTRL) and squeezed cells incubated in calcium-free media during and after squeezing (SQZD Ca^{2+}-free). Bar graph displaying quantification of fluorescence intensity for CD44 **(B)** and ABCB5 **(D)** across experimental groups. Bar graph displaying the mean fluorescence levels across 5 independent replicates per condition (≥20 cells per replicates). Bars represent mean ± SEM. Statistical significance was assessed using one-way ANOVA (two-sided). Asterisks indicate p-values, as follow: CD44, 2HRS p = 0.9829 (ns), 95% CI: [-12.90 to 6.581]; 4HRS p >0.9999 (ns), 95% CI: [-8.934, 10.55]; 24HRS p =0.9865 (ns), 95% CI: [-6.688, 12.80]; 48 HRS p >0.9999 (ns), 95% CI: [-9.420, 10.06]; 72 HRS p = 0.9934 (ns), 95% CI: [-13.97, 7.812]. ABCB5, 2HRS p = 0.5531 (ns), 95% CI: [-2.175, 9.236]; 4HRS p = 0.5043 (ns), 95% CI: [-6.671, 5.432]; 24HRS p =0.9945 (ns), 95% CI: [-4.130, 7.281]; 48 HRS p = 0.3257 (ns), 95% CI: [-1.539, 9.872]; 72 HRS p >0.9999 (ns), 95% CI: [-6.394, 6.364].

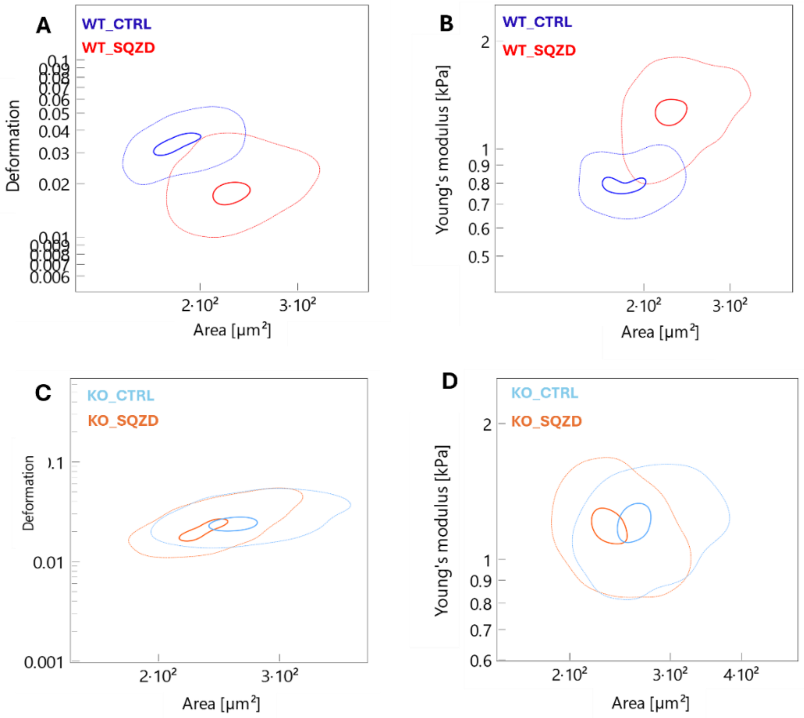

Supplementary Figure 12 PIEZO1 is required for post-constriction mechanical adaptation in melanoma cells. (A–B) Contour density plots showing the deformation **(A)** and apparent Young's modulus **(B)** of wild-type (WT) melanoma cells before (WT_CTRL, blue) and after (WT_SQZD, red) passing through a microfluidic constriction. **(C–D)** The same analysis for PIEZO1 knockout (KO) melanoma cells, comparing control (KO_CTRL, light blue) and constricted (KO_SQZD, orange) populations. Each contour plot represents the distribution of single-cell measurements (n > 1,000 cells per condition), with contour lines corresponding to 50% and 90% quantile density levels.

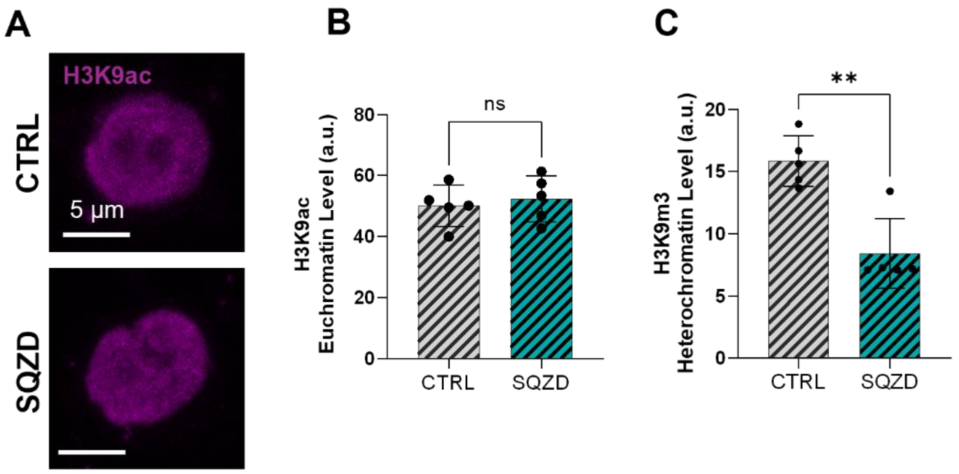

Supplementary Figure 13 Chromatin remodelling in SQZD-KO melanoma cells under mechanical constriction. (A) Representative staining of H3K27ac in CTRL and SQZ-KO melanoma cells. **(B)** Bar graph displaying the H3K9ac Euchromatin level in CTRL and SQZD-KO cells across 5 independent replicates per condition (≥20 cells per replicates). Bars represent mean ± SEM. Statistical significance was assessed using one-way ANOVA (two-sided). Asterisks indicate p-values, as follow: H3K9ac $p = 0.6292$ (ns) 95% CI: [-8.155, 12.69]. **(C)** Bar graph displaying the H3K9me3 Heterochromatin level in CTRL and SQZD-KO cells across 5 independent replicates per condition (≥20 cells per replicates). Statistical significance was assessed using one-way ANOVA (two-sided). Asterisks indicate H3K9me3 $p = 0.0014$ (ns) 95% CI: [-10.98, -3.849].

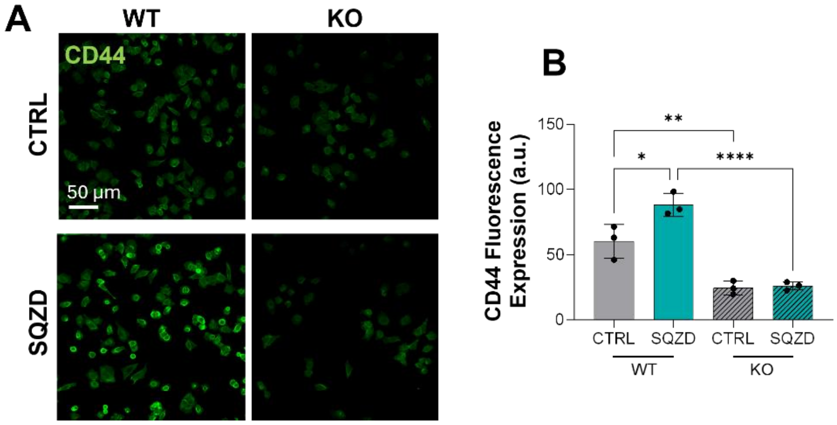

Supplementary Figure 14 CD44 Expression in Cells Escaping from Transwell. (A) Representative staining of CD44 in CTRL and SQZD melanoma cells (WT and KO). **(B)** Bar graph displaying the CD44 levels in CTRL and SQZD melanoma cells (WT and KO). Bar graph displaying the mean fluorescence levels across 3 independent replicates per condition (≥20 cells per replicates). Bars represent mean ± SEM. Statistical significance was assessed using one-way ANOVA (two-sided). Asterisks indicate p-values, as follow: WT_CTRL Vs WT_SQZD p = 0.0152 (*) 95% CI: [-50.06, -5.962]; WT_CTRL Vs KO_CTRL $p = 0.0038$ (**) 95% CI: [13.56, 57.67]; WT_SQZD Vs KO_SQZD CTRL $p < 0.0001$ (**) 95% CI: [40.21, 84.31].

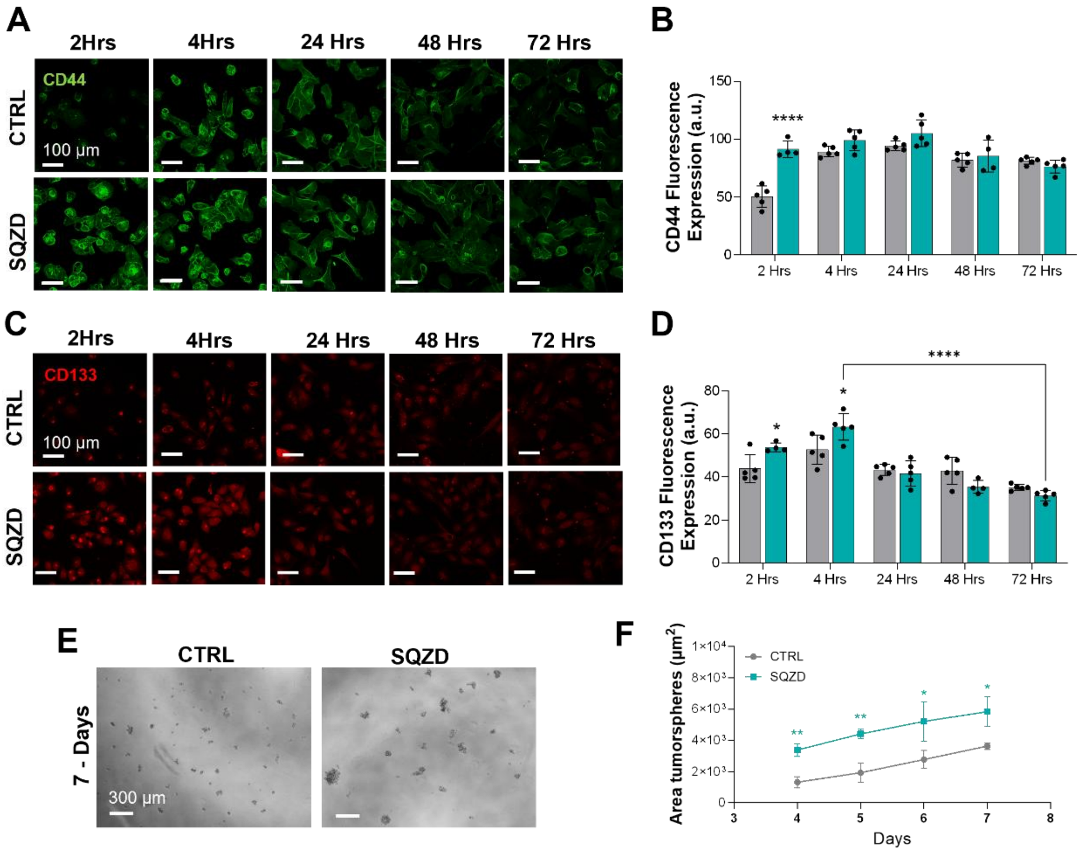

Supplementary Figure 15 Mechanical constriction induces stemness markers and tumorsphere formation in MDA-MB-231 cancer cell types. (A, C) Representative immunofluorescence images show expression of stemness markers CD44 (green) and CD133 (red) following mechanical constriction for MDA-MB-231 cells. **(B, D)** Bar graph showing the quantification of fluorescence intensity across 5 independent replicates per condition (≥20 cells per replicates). Bars represent mean ± SEM. Statistical significance was assessed using one-way ANOVA (two-sided). Asterisks indicate p-values, as follow: CD44, 2HRS p <0.0001 (****) 95% CI: [-59.70, -22.32]; CD133, 2HRS p = 0.0239 (*) 95% CI: [1.737, 17.95]; 4HRS p = 0.0326 (*) 95% CI: [1.123, 20.06]; 4HRS_SQZD Vs 72HRs_SQZD p <0.0001 (****) 95% CI: [21.16, 42.79]; **(E)** and **(F)** Representative brightfield images of tumorsphere formation and growth curves showing no significant differences between control and flow-only conditions, n = 3 independent replicates per condition. Bars represent mean ± SEM. Statistical significance was assessed using a two-sided unpaired t-test. Asterisks indicate as follow: 4 Days p = 0.0026 (**) 95% CI: [1200 to 2907]; 5 Days p = 0.0033 (**) 95% CI: [1382 to 3590]; 6 Days p = 0.0384 (**) 95% CI: [211.6 to 4658]; 7 Days p = 0.0174 (*) 95% CI: [635.5 to 3759].

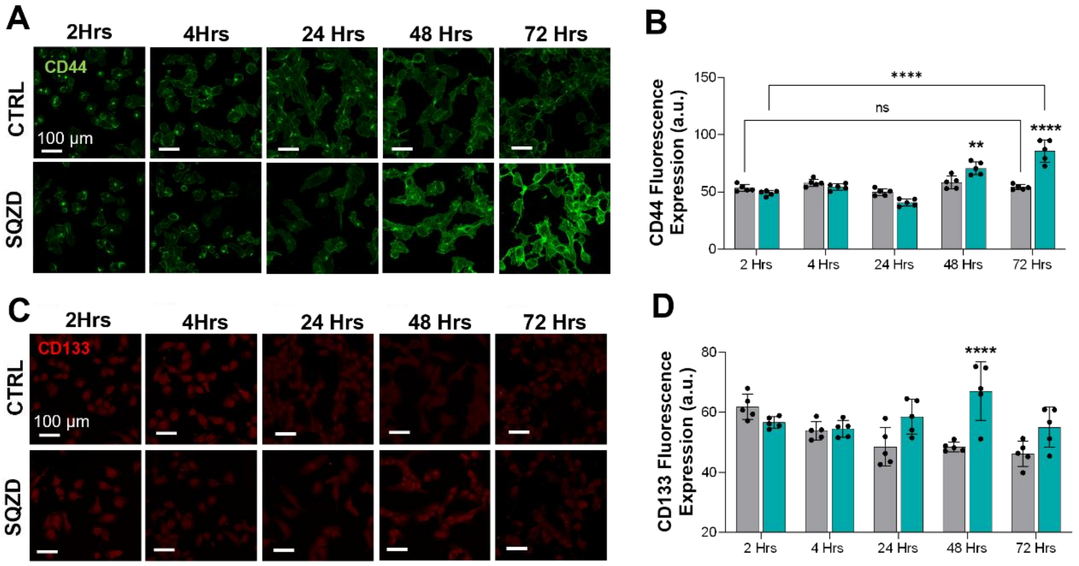

Supplementary Figure 16 Mechanical constriction induces stemness markers and tumorsphere formation in HT-1080 cancer cell types. (A, C) Representative immunofluorescence images show expression of stemness markers CD44 (green) and CD133 (red) following mechanical constriction for HT-1080 cells. **(B, D)** Bar graph showing the quantification of fluorescence intensity across 5 independent replicates per condition (≥20 cells per replicates). Bars represent mean ± SEM. Statistical significance was assessed using one-way ANOVA (two-sided). Asterisks indicate p-values, as follow: CD44, 72HRs_CTRL Vs 72HRs_SQZD p <0.0001 (****) 95% CI: [-41.55, -21.93]; CD133, 2HRS p = 0.0239 (*) 95% CI: [1.737, 17.95]; 2HRS_SQZD Vs 72HRs_SQZD p = 0.0326 (*) 95% CI: [1.123, 20.06]; 4HRS_SQZD Vs 72HRs_SQZD p <0.0001 (****) 95% CI: [-46.61, -26.99]. CD133, 48HRs p <0.0001 (****) 95% CI: -29.64, -7.486];

Supplementary Table 1 Indirect immunofluorescence labelling reagents

**Antibody**	**Dilution**	**Cat number**	**Host Species**	**Company**
**p-ERK**	1:200	9101S	Rabbit	Cell Signaling Technology
**p-AKT(308)**	1:500	77440S	Rabbit	Cell Signaling Technology
**NFkB**	1:200	8801S	Rabbit	Cell Signaling Technology
**p-p38**	1:200	8632S	Rabbit	Cell Signaling Technology
**YAP1**	1:500	AB225440	Rabbit	Abcam
**PKC**	1:200	MA1-157	Mouse	Thermo Fisher Scientific
**mTOR**	1:500	5048S	Rabbit	Cell Signaling Technology
**Anti-mouse AlexaFluor 647**	1:1000	4410S	Goat	Cell Signalling Technology
**anti-rabbit AlexaFluor 555**	1:1000	4413S	Goat	Cell Signalling Technology
**DAPI**	1:1500	D9542		Sigma-Aldrich
**Flash Phalloidin Green-488**	1:100	424201		BioLegend
