# Supplementary material for: Capillary constrictions prime cancer cell tumorigenicity through PIEZO1
Source: Nat Commun. 2025 Sep 1;16:8160. doi: 10.1038/s41467-025-63374-6 (PMC12402161; doi:10.1038/s41467-025-63374-6)
Supplement: Supplementary file 2 — Reporting Summary [file 41467_2025_63374_MOESM2_ESM.pdf]

Corresponding author(s): Kristopher Kilian
Last updated by author(s): Aug 6, 2025

Reporting Summary

Nature Portfolio wishes to improve the reproducibility of the work that we publish. This form provides structure for consistency and transparency in reporting. For further information on Nature Portfolio policies, see our Editorial Policies and the Editorial Policy Checklist.

Statistics

For all statistical analyses, confirm that the following items are present in the figure legend, table legend, main text, or Methods section.

n/a	Confirmed	
☐	☒	The exact sample size (*n*) for each experimental group/condition, given as a discrete number and unit of measurement
☐	☒	A statement on whether measurements were taken from distinct samples or whether the same sample was measured repeatedly
☐	☒	The statistical test(s) used AND whether they are one- or two-sided<br>*Only common tests should be described solely by name; describe more complex techniques in the Methods section.*
☒	☐	A description of all covariates tested
☒	☐	A description of any assumptions or corrections, such as tests of normality and adjustment for multiple comparisons
☐	☒	A full description of the statistical parameters including central tendency (e.g. means) or other basic estimates (e.g. regression coefficient) AND variation (e.g. standard deviation) or associated estimates of uncertainty (e.g. confidence intervals)
☐	☒	For null hypothesis testing, the test statistic (e.g. *F*, *t*, *r*) with confidence intervals, effect sizes, degrees of freedom and *P* value noted<br>*Give P values as exact values whenever suitable.*
☒	☐	For Bayesian analysis, information on the choice of priors and Markov chain Monte Carlo settings
☒	☐	For hierarchical and complex designs, identification of the appropriate level for tests and full reporting of outcomes
☒	☐	Estimates of effect sizes (e.g. Cohen's *d*, Pearson's *r*), indicating how they were calculated

Our web collection on statistics for biologists contains articles on many of the points above.

Software and code

Policy information about availability of computer code

Data collection	Zen Blue 2.5 (Zeiss) was used to acquire images from Zeiss LSM 800 confocal microscope.
Data analysis	Fiji-ImageJ (3) software was used to process images and extract quantitative data for analysis.<br>GraphPad Prism (9.5.0) software was used for statistical analysis and data visualization.

For manuscripts utilizing custom algorithms or software that are central to the research but not yet described in published literature, software must be made available to editors and reviewers. We strongly encourage code deposition in a community repository (e.g. GitHub). See the Nature Portfolio guidelines for submitting code & software for further information.

Data

Policy information about availability of data

All manuscripts must include a data availability statement. This statement should provide the following information, where applicable:

- Accession codes, unique identifiers, or web links for publicly available datasets
- A description of any restrictions on data availability
- For clinical datasets or third party data, please ensure that the statement adheres to our policy

The data generated in this work is included in the manuscript and supporting information. Data is available from the corresponding author upon reasonable request. The raw sequence data reported in this paper have been deposited in the Genome Sequence Archive (Genomics, Proteomics & Bioinformatics 2021) in National Genomics Data Center (Nucleic Acids Res 2022), China National Center for Bioinformation / Beijing Institute of Genomics, Chinese Academy of Sciences (GSA-

Human: HRA009642) that are publicly accessible at https://ngdc.cncb.ac.cn/gsa-human. The custom code used for the analysis of fluorescence marker signals from microscopy images is available on GitHub at https://github.com/ElvPan/VoronoiAndWatershedBased2DSegmentationWithCurvatureAnalysis. 'Source data are provided as a Source Data file.

Research involving human participants, their data, or biological material

Policy information about studies with human participants or human data. See also policy information about sex, gender (identity/presentation), and sexual orientation and race, ethnicity and racism.

Reporting on sex and gender	*Use the terms sex (biological attribute) and gender (shaped by social and cultural circumstances) carefully in order to avoid confusing both terms. Indicate if findings apply to only one sex or gender; describe whether sex and gender were considered in study design; whether sex and/or gender was determined based on self-reporting or assigned and methods used. Provide in the source data disaggregated sex and gender data, where this information has been collected, and if consent has been obtained for sharing of individual-level data; provide overall numbers in this Reporting Summary. Please state if this information has not been collected. Report sex- and gender-based analyses where performed, justify reasons for lack of sex- and gender-based analysis.*
Reporting on race, ethnicity, or other socially relevant groupings	*Please specify the socially constructed or socially relevant categorization variable(s) used in your manuscript and explain why they were used. Please note that such variables should not be used as proxies for other socially constructed/relevant variables (for example, race or ethnicity should not be used as a proxy for socioeconomic status). Provide clear definitions of the relevant terms used, how they were provided (by the participants/respondents, the researchers, or third parties), and the method(s) used to classify people into the different categories (e.g. self-report, census or administrative data, social media data, etc.) Please provide details about how you controlled for confounding variables in your analyses.*
Population characteristics	*Describe the covariate-relevant population characteristics of the human research participants (e.g. age, genotypic information, past and current diagnosis and treatment categories). If you filled out the behavioural & social sciences study design questions and have nothing to add here, write "See above."*
Recruitment	*Describe how participants were recruited. Outline any potential self-selection bias or other biases that may be present and how these are likely to impact results.*
Ethics oversight	*Identify the organization(s) that approved the study protocol.*

Note that full information on the approval of the study protocol must also be provided in the manuscript.

Field-specific reporting

Please select the one below that is the best fit for your research. If you are not sure, read the appropriate sections before making your selection.

[x] Life sciences [ ] Behavioural & social sciences [ ] Ecological, evolutionary & environmental sciences

For a reference copy of the document with all sections, see nature.com/documents/nr-reporting-summary-flat.pdf

Life sciences study design

All studies must disclose on these points even when the disclosure is negative.

Sample size	No specific hypothesis or calculation of sample sizes was performed. Imaging data for each group were collected from at least five independent image regions (containing multiple cells) to represent the population under each condition. For in vivo studies, sample sizes (n = 5) were chosen to minimize animal use while enabling the detection of meaningful differences in tumorigenicity between groups. All key experiments were independently repeated at least three times, and similar trends were observed across biological replicates. The observed consistency in experimental outcomes and statistical significance across replicates supports both the reproducibility and adequacy of the chosen sample sizes. No findings reported in the study failed to replicate.
Data exclusions	No data were excluded from the in vitro studies.
Replication	For in vitro experiments, a minimum of three and a maximum of five independent biological replicates was used, unless otherwise stated, consistent with standards for cellular and molecular assays.
Randomization	All cell samples were randomly allocated to each experimental group. For in vivo experiments, animals were age- and sex-matched and assigned to groups based on predefined experimental design. Experimental procedures were standardized across all groups to minimize potential bias.
Blinding	The same investigators both designed and performed experiments and data analysis, therefore no blinding concerning sample identity.

Reporting for specific materials, systems and methods

We require information from authors about some types of materials, experimental systems and methods used in many studies. Here, indicate whether each material, system or method listed is relevant to your study. If you are not sure if a list item applies to your research, read the appropriate section before selecting a response.

Materials & experimental systems

n/a	Involved in the study
☐	☒ Antibodies
☐	☒ Eukaryotic cell lines
☒	☐ Palaeontology and archaeology
☐	☒ Animals and other organisms
☒	☐ Clinical data
☒	☐ Dual use research of concern
☒	☐ Plants

Methods

n/a	Involved in the study
☒	☐ ChIP-seq
☒	☐ Flow cytometry
☒	☐ MRI-based neuroimaging

Antibodies

Antibodies used

Primary antibodies:
Anti-CD44 (ThermoFisher Scientific Australia, Cat. Number a),
Anti-p75 NGF Receptor antibody [8J2] CD271(abcam, Cat. Number AB245134)
Anti-ABCB5 (Novus Biologicals, Cat. Number NBP1-77687), PRDM14 (Abclonal, Cat. Number A5543)
Anti-PIEZO1 (ThermoFisher Scientific Australia, Cat. Number MA5-32876)
Anti-H3K9m3 (Cell Signalling, Cat. Number D4W1U)
p-ERK (Cell Signaling Technology)
p-AKT(308) (Cell Signaling Technology)
NFkB (Cell Signaling Technology)
p-p38(Cell Signaling Technology)
YAP1 (Abcam)
PKC (Thermo Fisher Scientific)
mTOR (Signaling Technology)

Secondary antibodies:
Goat anti-Mouse IgG (H+L) Secondary Antibody, DyLight™ 488 from Thermo Fischer Scientific, Cat. Number 35502
Anti-Rabbit IgG (H+L), CF™ 647 antibody produced in goat from Sigma-Aldrich, Cat number SAB4600184
Hoescht 33342 (ThermoFisher Scientific; H3570).
Anti-mouse AlexaFluor 647 (Cell Signalling Technology)
anti-rabbit AlexaFluor 555 (Cell Signalling Technology)
DAPI (Sigma-Aldrich)
Flash Phalloidin Green-488(BioLegend)

Validation

Control conditions without primary antibody were used to acquire background signal. Validations were available from manufacturers.

Eukaryotic cell lines

Policy information about cell lines and Sex and Gender in Research

Cell line source(s)

A375-MA2 were obtained from ATCC (Cat. Number CRL-3223). PIEZO1 knockout cells were generated using CRISPR/Cas9 editing, as described in the manuscript. A375-Luc2 human melanoma cells used for the in vivo model were obtained from ATCC (Cat. Number CRL-1619-LUC2). HUVECs were purchased from Lonza (Cat. Number C2519A)

Authentication

375-MA2, A375-Luc2 and HUVEC cells have been authenticated by ATCC.

Mycoplasma contamination

All cell lines used in this study tested negative for mycoplasma contamination.

Commonly misidentified lines
(See ICLAC register)

The cells used in this study are not present in the ICLAC register.

Animals and other research organisms

Policy information about studies involving animals; ARRIVE guidelines recommended for reporting animal research, and Sex and Gender in Research

Laboratory animals

BALB/c nude mice, 7 weeks of age. from Australian BioResources (ABR) and Ozgene ARC Australia

Wild animals

n/a

Reporting on sex	Female mice were used in study
Field-collected samples	Mice were housed in groups of five per cage under specific pathogen-free (SPF) conditions, maintained at 21–23 °C with 40–60% relative humidity and a 12-hour light/dark cycle. Food and water were provided ad libitum.
Ethics oversight	UNSW Animal Care & Ethics Committee (ACEC 22-85B); National Health and Medical Research Council Statement on Animal Experimentation, the re-quirements of New South Wales State Government legislation, and the rules for animal experimen-tation of the Biological Testing Facility of the Garvan Institute and the Victor Chang Cardiac Re-search Institute (protocol #24_25).

Note that full information on the approval of the study protocol must also be provided in the manuscript.

Plants

Seed stocks	*Report on the source of all seed stocks or other plant material used. If applicable, state the seed stock centre and catalogue number. If plant specimens were collected from the field, describe the collection location, date and sampling procedures.*
Novel plant genotypes	*Describe the methods by which all novel plant genotypes were produced. This includes those generated by transgenic approaches, gene editing, chemical/radiation-based mutagenesis and hybridization. For transgenic lines, describe the transformation method, the number of independent lines analyzed and the generation upon which experiments were performed. For gene-edited lines, describe the editor used, the endogenous sequence targeted for editing, the targeting guide RNA sequence (if applicable) and how the editor was applied.*
Authentication	*Describe any authentication procedures for each seed stock used or novel genotype generated. Describe any experiments used to assess the effect of a mutation and, where applicable, how potential secondary effects (e.g. second site T-DNA insertions, mosiacism, off-target gene editing) were examined.*
